# Myeloid-derived suppressor cells are associated with impaired Th1 and Th17 responses and severe pulmonary paracoccidioidomycosis which is reversed by anti-Gr1 therapy

**DOI:** 10.3389/fimmu.2023.1039244

**Published:** 2023-01-26

**Authors:** Nycolas Willian Preite, Valéria de Lima Kaminski, Bruno Montanari Borges, Vera Lúcia Garcia Calich, Flávio Vieira Loures

**Affiliations:** ^1^ Institute of Science and Technology, Federal University of São Paulo, São José dos Campos, São Paulo, Brazil; ^2^ Department of Immunology, Institute of Biomedical Sciences, University of São Paulo, São Paulo, Brazil

**Keywords:** paracoccidioidomycosis (PCM), MDSC (myeloid-derived suppressor cell), immunoregulation, fungal infection, lung immune cells

## Abstract

Previous studies on paracoccidioidomycosis (PCM), the most prevalent systemic mycosis in Latin America, revealed that host immunity is tightly regulated by several suppressive mechanisms mediated by tolerogenic plasmacytoid dendritic cells, the enzyme 2,3 indoleamine dioxygenase (IDO-1), and regulatory T-cells (Tregs). IDO-1 orchestrates local and systemic immunosuppressive effects through the recruitment and activation of myeloid-derived suppressor cells (MDSCs), a heterogeneous population of myeloid cells possessing a potent ability to suppress T-cell responses. However, the involvement of MDSCs in PCM remains uninvestigated. The presence, phenotype, and immunosuppressive activity of MDSCs were evaluated at 96 h, 2 weeks, and 8 weeks of pulmonary infection in C57BL/6 mice. Disease severity and immune responses were assessed in MDSC-depleted and nondepleted mice using an anti-Gr1 antibody. Both monocytic-like MDSCs (M-MDSCs) and polymorphonuclear-like MDSCs (PMN-MDSCs) massively infiltrated the lungs during *Paracoccidioides brasiliensis* infection. Partial reduction of MDSC frequency led to a robust Th1/Th17 lymphocyte response, resulting in regressive disease with a reduced fungal burden on target organs, diminishing lung pathology, and reducing mortality ratio compared with control IgG2b-treated mice. The suppressive activity of MDSCs on CD4 and CD8 T-lymphocytes and Th1/Th17 cells was also demonstrated *in vitro* using coculture experiments. Conversely, adoptive transfer of MDSCs to recipient *P. brasiliensis*-infected mice resulted in a more severe disease. Taken together, our data showed that the increased influx of MDSCs into the lungs was linked to more severe disease and impaired Th1 and Th17 protective responses. However, protective immunity was rescued by anti-Gr1 treatment, resulting in a less severe disease and controlled tissue pathology. In conclusion, MDSCs have emerged as potential target cells for the adjuvant therapy of PCM.

## Introduction

Paracoccidioidomycosis (PCM) is a systemic chronic mycosis characterized by granulomatous lesions caused by the thermally dimorphic fungi *Paracoccidioides brasiliensis* and *Paracoccidioides lutzii* as well as the more recently described *Paracoccidioides americana, Paracoccidioides restrepiensis*, and *Paracoccidioides venezuelensis* (1). PCM is endemic, possessing a high incidence rate in several Latin American countries such as Brazil, Colombia, Venezuela, Ecuador, and Argentina, with a non-uniform distribution among species that is more prominent in some regions than in others (1–5). PCM is associated with the highest mortality rate (1.45 per million inhabitants) among systemic mycoses; furthermore, it is considered an occupational disease that leads to serious health and socioeconomic problems because it can affect the ability of farmers to perform their activities (3). Infection occurs following the inhalation of mycelial fragments or conidia into the lungs and frequently leads to pulmonary disorders owing to primary infection progression or reactivation of a latent focus (6–10).

In humans and murine models of PCM, disease resistance has been associated with the secretion of interferon gamma (IFN-γ) and other antigens specific to *Paracoccidioides* spp T helper 1 (Th1)-secreted cytokines, whereas impaired Th1 and the prevalent secretion of Th2 cytokines have been correlated with systemic and progressive disease (11–14). However, the importance of Th17 immunity is not well understood. For instance, IL-17-expressing cells have been observed in the cutaneous and mucosal lesions of patients with PCM and have been associated with granuloma organization (14). Moreover, recently, diverse patterns of T-cell responses in *P*. *brasiliensis*-infected individuals reportedly lead to different clinical manifestations. Resistance to infection observed in asymptomatic individuals was shown to be mediated by a predominant Th1 response, which is responsible for macrophage activation. Nevertheless, PCM patients with mild forms of the disease (mainly the chronic form) showed reactivity to paracoccidioidin, indicating the presence of a Th1 response, albeit one that is insufficient to contain the infection. The most severe form of the disease, the juvenile form, presents with a prevalent Th2/Th9 response and an enhanced antibody response. However, severe cases of the chronic form of the disease also present a predominant Th2 response. Prominent Th17 immunity associated with important participation of Th1 cells was also described in the chronic inflammatory response characteristic of the adult form of the disease (15).

Our previous studies revealed that host immunity is tightly regulated by several suppressive mechanisms mediated by tolerogenic plasmacytoid dendritic cells (pDCs), the potent immunoregulatory enzyme indoleamine-2,3-dioxygenase (IDO-1), and regulatory T-cells (Tregs) (13, 16–19). Several studies have evaluated the role of Treg cells in the severity of PCM, revealing either deleterious or protective effects depending on the experimental model employed (20, 21). The beneficial effects of Treg cell depletion performed by treatment of C57BL/6DTR/eGFP (DEREG) mice with diphtheria toxin on established PCM were mediated by the rescue of protective immunity and prevention of fatal disease outcomes (22). Moreover, immunosuppression observed in patients with PCM has been associated with elevated numbers of FoxP3^+^ Treg cells in their lesions and blood (23, 24). We demonstrated that the tolerogenic activity of pDCs enhanced the severity of pulmonary PCM mediated by the concerted action of IDO-1 and Treg cells (16). As previously reported in candidiasis and aspergillosis (25), an IDO-1-mediated immunomodulatory function has also been described in pulmonary PCM (16, 18, 19, 26).

IDO-1 orchestrates local and systemic immunosuppressive effects through the recruitment and activation of myeloid-derived suppressor cells (MDSCs), which is a heterogeneous population of myeloid cells having a potent ability to suppress T-cell responses. These cells regulate immune responses and tissue repair in healthy individuals, with their population rapidly expanding during inflammation and infection. When reaching target organs that exhibit a diverse range of cytokines, chemokines, and other inflammatory mediators, MDSCs sense and adapt their suppressive behavior by acquiring different suppressive mechanisms, such as those mediated by the production of IDO-1 or IL-10, or the expression of programmed death-ligand 1 (PD-L1), an immunological checkpoint inhibitor (27–29).

MDSCs comprise morphologically distinct subsets of monocyte-like (monocytic-MDSCs (M-MDSCs)) and neutrophil-like (polymorphonuclear-MDSCs (PMN-MDSC)) cells. PMN-MDSCs are defined as CD11b^+^Ly6G^+^Ly6C^low^, whereas M-MDSCs as CD11b^+^Ly6G^−^Ly6C^hi^, both of which are implicated in various aspects of immune regulation in diseases that involve chronic inflammation, such as neoplasias, infections, autoimmune diseases, and other pathologies (30). Recent studies have highlighted the emerging role of MDSCs in pulmonary diseases, including tuberculosis and COVID-19; their presence has been correlated with the development of chronic infection owing to their immunoregulatory properties (31–37). The immunosuppressive mechanisms of MDSCs restrict both the proliferation and release of cytokines by effector CD4^+^ and CD8^+^ T-cells, while inducing apoptotic cell death (29).

Few studies have shown the involvement of MDSCs in fungal infections; however, some groups have demonstrated the induction of PMN-MDSCs following infection with *Candida albicans* or *Aspergillus fumigatus* (38). Induction of MDSCs in fungal infections was further observed to be dependent on pathways downstream of dectin-1/CARD9 signaling, notably generation of reactive oxygen species (ROS), caspase-8 activity, and IL-1β production. Moreover, Th17-polarized immunity, which is generally required for protection against fungal infections, causes excessive inflammatory reactions that are modulated by MDSCs. Although several immunoregulatory mechanisms and mediators are involved in host responses to PCM, such as IDO-1 production (16, 18, 19), TLRs, dectin-1, iNOS activation (39–42), and IL-10 and TGF-β secretion (43, 44), the involvement of MDSCs in the regulatory mechanisms that control pulmonary PCM has not yet been investigated.

MDSC depletion has been achieved in murine models of tumor and infectious diseases through the use of pharmacological compounds or anti-Gr1^+^ antibodies to better understand the role of these immunosuppressive cells (34, 45–47). Anti-Gr1 antibodies can bind to two molecules of the Ly6 superfamily, Ly6G and Ly6C, which are both present on the MDSC surface. Notably, anti-Gr1 therapy prevented MDSC accumulation and therefore promoted less pulmonary disease development and increased mice survival in infection models of tuberculosis (34) or cryptococcosis (47). Here, we demonstrated a robust pulmonary influx of MDSCs into the lungs of *P. brasiliensis*-infected mice 96 h, 2 weeks, and 8 weeks after infection. Using an anti-Gr1 antibody, we demonstrated that partial reduction of the number of MDSCs led to robust Th1/Th17 lymphocyte responses. In the current study, the more effective immune response resulted in regressive disease with reduced fungal burden, lung pathology, and mortality ratios. The suppressive effects of MDSCs on Th1/Th17 expansion were confirmed using *in vitro* coculture experiments. Furthermore, transfer of MDSCs to recipient *P. brasiliensis*-infected mice resulted in more severe disease. Altogether, our data demonstrated for the first time the crucial role of MDSCs in the control of pulmonary PCM.

## Methods

### Ethics statement

The experiments were performed in strict accordance with the Brazilian Federal Law 11,794 establishing procedures for the scientific use of animals, and the State Law establishing the Animal Protection Code of the State of São Paulo. All efforts were made to minimize animal suffering. The procedures were approved by the Ethics Committee on Animal Experiments of the Federal University of São Paulo (Protocol N° 8294230519).

### Mice

Eight- to 12-week-old male C57BL6/J WT mice was bred as specific pathogen-free mice at the Center for the Development of Experimental Models for Biology and Medicine, Federal University of São Paulo - CEDEME-UNIFESP, and kept in the Facility of the Institute of Science and Technology of the Federal University of São Paulo - ICT-UNIFESP.

### Fungal strain and infection

Virulent *P. brasiliensis* 18 (Pb18 isolate) yeast cells were maintained by weekly cultivation in Fava Netto culture medium at 37°C and used on days 7–8 of culture. The viability of fungal cells, which was always higher than 95%, was determined using Janus Green B vital dye (Merck). Mice were anesthetized and subjected to intratracheally (it.) infection as previously described (48). Briefly, after intraperitoneal (ip.) injection of ketamine (90 mg/kg) and xylazine (10 mg/kg), animals were infected with 1 × 10^6^ yeast cells in 50 μL PBS by surgical (it.) inoculation, which allowed direct dispensing of the fungal cells into the lungs.

### Depletion of MDSCs

C57BL/6 WT mice were infected with 1 × 10^6^ P*. brasiliensis* yeast cells. After 24 h, 1 week, and 7 weeks of infection, a group of mice received intraperitoneal injections of anti-Gr1 antibody (clone RB6-8C5, BioXCell), or rat IgG2b isotype control antibody (200 µg/dose). Injections were administered every 48 h. After 96 h, 2 weeks, and 8 weeks of infection, the mice were euthanized. The lung, liver, and spleen were collected to analyze the disease severity, through evaluation of colony-forming unit (CFU) counts and the levels of cytokines by ELISA. The depletion of MDSCs and other leukocytes in the lungs was monitored using flow cytometric analysis. The diagram in the **Supplementary Figure 1** illustrates the anti-Gr1 treatments used to analyze disease severity after infection.

### CFU assays, mortality rates, and histological analysis

The number of viable yeast cells in the lungs, liver, and spleen was determined by counting the number of CFU, as previously described (49). Mortality studies were performed using groups of 10–11 mice. Deaths were registered daily. For histological examinations, 5-μm-thick tissue sections were stained with hematoxylin-eosin (HE) and silver (Grocott stain) for lesions and fungal evaluation, respectively. Morphometric analysis was performed using a Nikon DXM 1200c camera and Nikon NIS AR 2.30 software. The areas of lesions were measured (in μm^2^) in 10 microscopic fields per slide in 5 mice per group, as previously described (50). Results are expressed as the mean ± SEM of the total lesion area for each mouse.

### Lung infiltrating leucocytes and flow cytometric analysis

The lungs were collected from anti-Gr1-treated and untreated *P. brasiliensis*-infected C57BL/6 WT mice after 96 h, 2 weeks, and 8 weeks of infection. The tissues were digested enzymatically, and lung leukocytes were prepared as previously described (41). For cell surface staining, lung cells were suspended at 1 × 10^6^ cells/mL in staining buffer. The Fc receptors were blocked with unlabeled anti-CD16/32 (eBioscience) and stained for 30 min at 4°C with fluorophore-conjugated antibodies. The following phenotypes were used to determine leukocyte populations: CD45^+^CD11b^+^Ly6G^+^Ly6C^low^ for PMN-MDSCs; CD45^+^CD11b^+^Ly6G^-^Ly6C^hi^ for M-MDSCs; CD45^+^CD11b^+^F4/80^+^ for macrophages; CD45^+^CD11b^+^CD11c^+^F4/80^-^ for dendritic cells; CD45^+^CD11b^+^Ly6G^hi^ for neutrophils; CD45^+^CD4^+^ and CD45^+^CD4^+^CD25^+^ for CD4 T-lymphocytes; and CD45^+^CD8^+^ and CD45^+^CD8^+^CD69^+^ for CD8^+^ T-lymphocytes. Intracellular staining was performed to identify IDO-1^+^, nitrotyrosine (NT^+^), and IL-10^+^ MDSCs. Intracellular staining was also used to identify Th1 (CD4^+^IFN-y^+^), Th2 (CD4^+^IL-4^+^), Th17 (CD4^+^IL-17^+^), Tc1 (CD8^+^IFN-y^+^), Tc2 (CD8^+^ IL-4^+^), and Tc17 (CD8^+^IL-17^+^) lymphocytes. Cells were run on FACS Lyric (BD Biosciences), and a minimum of 50 000 events were acquired using the FACSuite software (BD Biosciences). Analysis was performed using the FlowJo software (Tree Star).

### *In vitro* generation of BM-MDSCs

Bone marrow MDSCs (BM-MDSCs) were generated from BM cells of C57BL/6 WT mice as previously described (51). Briefly, BM cells were flushed out from within long bones (femurs and tibias) using a 1 mL syringe and RPMI-1640 medium (Sigma-Aldrich) supplemented with 3% fetal bovine serum (FBS, Sigma-Aldrich). Red blood cells (RBC) were lysed using RBC lysis buffer (BioLegend). After RBC lysis, 7 × 10^5^ white blood cells were seeded in cell culture bottles containing Dulbecco’s modified Eagle medium (DMEM, Sigma-Aldrich) supplemented with 10% FBS in the presence of recombinant murine IL-6 and GM-CSF (both at 10 ng/mL; Biolegend) and cultured for 3 d at 37°C and 5% CO_2_; the medium was changed after 48 h. Subsequently, BM-MDSCs were separated from other cell populations in the culture using the myeloid-derived suppressor cell isolation kit (Miltenyi Biotec), following the manufacturer’s instructions. The purity and characterization of isolated MDSCs were evaluated using flow cytometry.

### *In vitro* generation of BM-dendritic cells

Bone marrow dendritic cells (BM-DCs) were differentiated from BM cells of C57BL/6 WT mice as previously described (13). Briefly, BM cells were flushed out from within long bones (femurs and tibias) using a 1 mL syringe and RPMI-1640 supplemented with 3% FBS. RBCs were lysed using RBC lysis buffer according to the manufacturer’s instructions, and 1 × 10^6^ cells/mL were seeded in cell culture bottles containing RPMI supplemented with 10% FBS in the presence of 20 ng/mL GM-CSF (BioLegend) and 2 ng/mL IL-4 (BioLegend). BM cells were cultured for 5 d at 37°C and 5% CO_2_. On the sixth day of culture, CD11c^+^ cells were isolated by magnetic separation using the CD11c MicroBeads UltraPure kit (Miltenyi Biotec) according to the manufacturer’s instructions. BM-DCs were then challenged with *P. brasiliensis* overnight at a ratio of 1:50 (yeast:DCs) and cocultured with T-lymphocytes and BM-MDSCs for 4 d.

### Isolation of lung-MDSCs

To investigate the activity of MDSCs obtained from infected animals, cells were recovered from the lungs of C57BL/6 WT mice 2 weeks after infection with *P. brasiliensis* yeasts. Lung infiltrating leukocytes were obtained as described in the section *Lung Infiltrating Leucocytes and Flow Cytometric Analysis* above, whereas lung MDSCs were isolated using the Myeloid-Derived Suppressor Cell Isolation Kit (Miltenyi Biotec). Both the purity and characterization of isolated MDSCs were evaluated by flow cytometry. The Purity of MDSCs^+^ cells used was always higher than 90%.

### Immunosuppressive activity of MDSCs on T-lymphocytes

For T-cell isolation, spleens from *naïve* C57BL/6 WT mice were homogenized to generate single-cell suspensions of splenocytes. After lysis using RBC buffer, T-cell populations were separated using the Pan T Cell Isolation Kit (Miltenyi Biotec) following the manufacturer’s instructions. To evaluate the proliferation of T-lymphocytes, cells were stained with carboxyfluorescein succinimidyl ester (CellTrace™ CFSE Cell Proliferation Kit, Invitrogen) according to the manufacturer’s instructions. Subsequently, 1 × 10^6^ T-lymphocytes were activated with 1 µg/mL anti-CD3 and anti-CD28 monoclonal antibodies (BioLegend) and cocultured at 37°C and 5% CO_2_ for 4 d in the presence or absence of 1 × 10^5^ BM-MDSCs (1:10; MDSC:T cell ratio) or lung-isolated MDSCs (1:10 and 1:5; MDSC:T cell ratios) per well in a 96-well U-bottom plate. BM-MDSCs were previously challenged with 4 × 10^3^ *P. brasiliensis* (1:50; yeast:MDSC ratio). After coculture, T-cells were stained with fluorochrome-conjugated anti-mouse antibodies to identify both CD4^+^ and CD8^+^ T-cell populations. The proliferation of T-cells was defined according to the CFSE dilution and assessed by flow cytometry (52). To assess the influence of MDSCs on Th cell profiles, T-cells isolated from spleens of *naïve* C57BL/6 mice were cocultured with BM-MDSCs for 4 d at a ratio of 1:10 (MDSC:T-cell) in RPMI-1640 medium containing 10% FBS at 37°C and 5% CO_2_. Importantly, T-cells were activated for 24 h by BM-DCs previously challenged with *P. brasiliensis* yeasts at a ratio of 1:10 (DC:T-cells). Subsequently, 1 × 10^5^ MDSCs were cocultured with 1 × 10^6^ T-lymphocytes per well in 96-well U-bottom plates. After 4 d of coculture, extracellular and intracellular staining of T-lymphocytes was performed and analyzed by flow cytometry.

### Adoptive transfer of MDSCs

Donor mice were infected with *P. brasiliensis via* the it. route. After two weeks of infection, the lungs were obtained and MDSCs were isolated as previously described in *Isolation of Lung-MDSCs* section. Then, the lung MDSCs were transferred into the infected recipient mice (2 × 10^6^ cells/mouse) through the it. route. Seven weeks before adoptive cell transfer, recipient mice were it. infected with *P. brasiliensis*. Seven days after the transfer of lung-MDSCs, mice were euthanized, and the organs were collected to analyze the severity of disease through the evaluation of CFU counts and levels of cytokines by ELISA.

### Cytokines

After 96 h, 2 weeks, and 8 weeks of infection, lungs from *P. brasiliensis*-infected mice were aseptically collected, disrupted, and the obtained supernatants were stored at -80°C. The levels of IFN-γ, TNF-α, IL-2, IL-1β, IL-4, IL-6, IL-12, IL-17, and IL-23 were determined in lung homogenates using capture ELISA kits purchased from BioLegend. The levels of IL-10 and IL-22 were analyzed using ELISA kits from eBioscience, whereas those of TGF-β using kits from Invitrogen. Plates were read using a spectrophotometric plate reader (VersaMax, Molecular Devices).

### Statistical analysis

Data are expressed as the M ± SEM. Differences between groups were analyzed by non-paired Student’s t-test or analysis of variance (ANOVA) followed by the Tukey test. Differences between survival times were determined with the log-rank test. Data were analyzed using GraphPad Prism 9 software (GraphPad Prism Software, Inc.). p values ≤ 0.05 were considered significant.

## Results

### Characterization of MDSCs in the lungs of *P. brasiliensis*-infected mice

**Figure 1:**
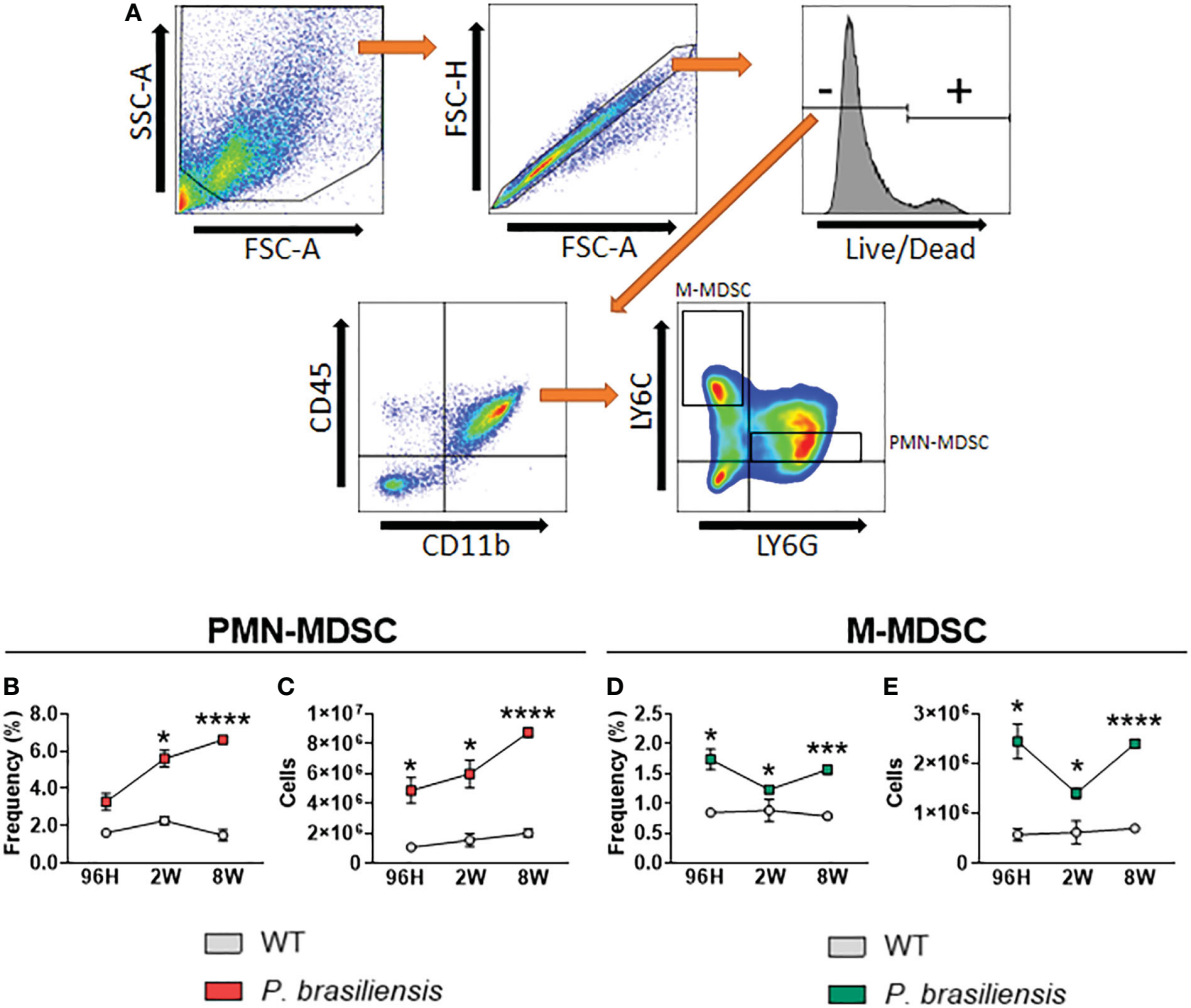
MDSC response to *P. brasiliensis* infection. C57BL/6 mice were infected through the intratracheal route with 1 × 10^6^ *P. brasiliensis* yeasts. Lungs were obtained after 96 hours, 2 weeks, and 8 weeks of infection, and compared with lungs from uninfected control animals. Characterization of subpopulations was performed using specific antibodies conjugated to fluorochromes. **(A)** Cells were gated by FSC/SSC analysis, and populations of PMN-MDSCs (CD45^+^CD11b^+^LY6G^+^LY6C^low^) and M-MDSCs (CD45^+^CD11b^+^LY6G^-^LY6C^hi^) were represented in frequency and absolute numbers. **(B-E)** Data represent three independent experiments with 3-4 mice each. For comparisons between two groups, the mean ± SEM was obtained and analyzed by the unpaired Student’s *t*-test. Differences were considered significant when *p<0.05; ***p<0.001 and ****p<0.0001.

To assess the involvement of MDSCs and their subtypes during pulmonary PCM, we infected C57BL/6 mice with 1 × 10^6^ P*. brasiliensis* through the intratracheal route. After 96 h, 2 weeks, and 8 weeks of infection, animals were euthanized, their lungs removed, and lung infiltrating leukocytes were obtained. We then subjected cell subpopulations to multiparametric flow cytometric analysis. After labeling with specific antibodies, we used gate strategies to define cell subpopulations (**Figure 1A**). Leukocytes were first gated as CD45^+^CD11b^+^ and later classified for the expression of Ly6G and Ly6C; PMN-MDSCs were classified as Ly6G^+^Ly6C^low^, whereas M-MDSCs were classified as Ly6G^-^Ly6C^hi^. Regarding the PMN-MDSC subset at 96 h of infection, although we did not observe any statistically significant differences in frequency (*p* = 0.054), we detected an increased number of cells in *P. brasiliensis*-infected mice than in the control mice. At 2 and 8 weeks postinfection, we observed an increased frequency and number of PMN-MDSCs in the lungs of infected mice than in those of their uninfected counterparts (**Figures 1B, C**). The time-point graphs in **Figures 1B, C** depict the increased influx of PMN-MDSCs during the course of infection. Importantly, we found that both the frequency and number of this immunosuppressive cell population were increased during the course of disease, reaching higher numbers in the eighth week of infection. We obtained similar results for the monocytic suppressor population. We observed an increase in both the frequency and number of M-MDSCs at all postinfection time points studied compared with that in uninfected mice (**Figures 1D, E**). Moreover, similar to that for PMN-MDSCs, we detected higher frequencies and numbers of M-MDSCs during the chronic phase of infection compared with those in the acute phase of infection.

### Expression of immunosuppressive molecules by PMN-MDSCs during *P. brasiliensis* infection

To better characterize the function of MDSCs in pulmonary PCM, we determined the expression of immunosuppressive molecules associated with their regulatory activity (29). After labeling with specific antibodies, we evaluated each MDSC subpopulation for the expression of CD274 (PD-L1), B7-H4, IDO-1, IL-10, and nitrotyrosine (NT). Both B7-H4 and PD-L1 are important members of the B7 family of proteins, and their expression in MDSC cell membranes has been previously associated with tumor pathologies (53, 54). Particularly, the interaction of PD-1 on activated T-cells with PD-L1-expressing MDSCs has been reported to inhibit the activation and proliferation of lymphocytes (55).

**Figure 2:**
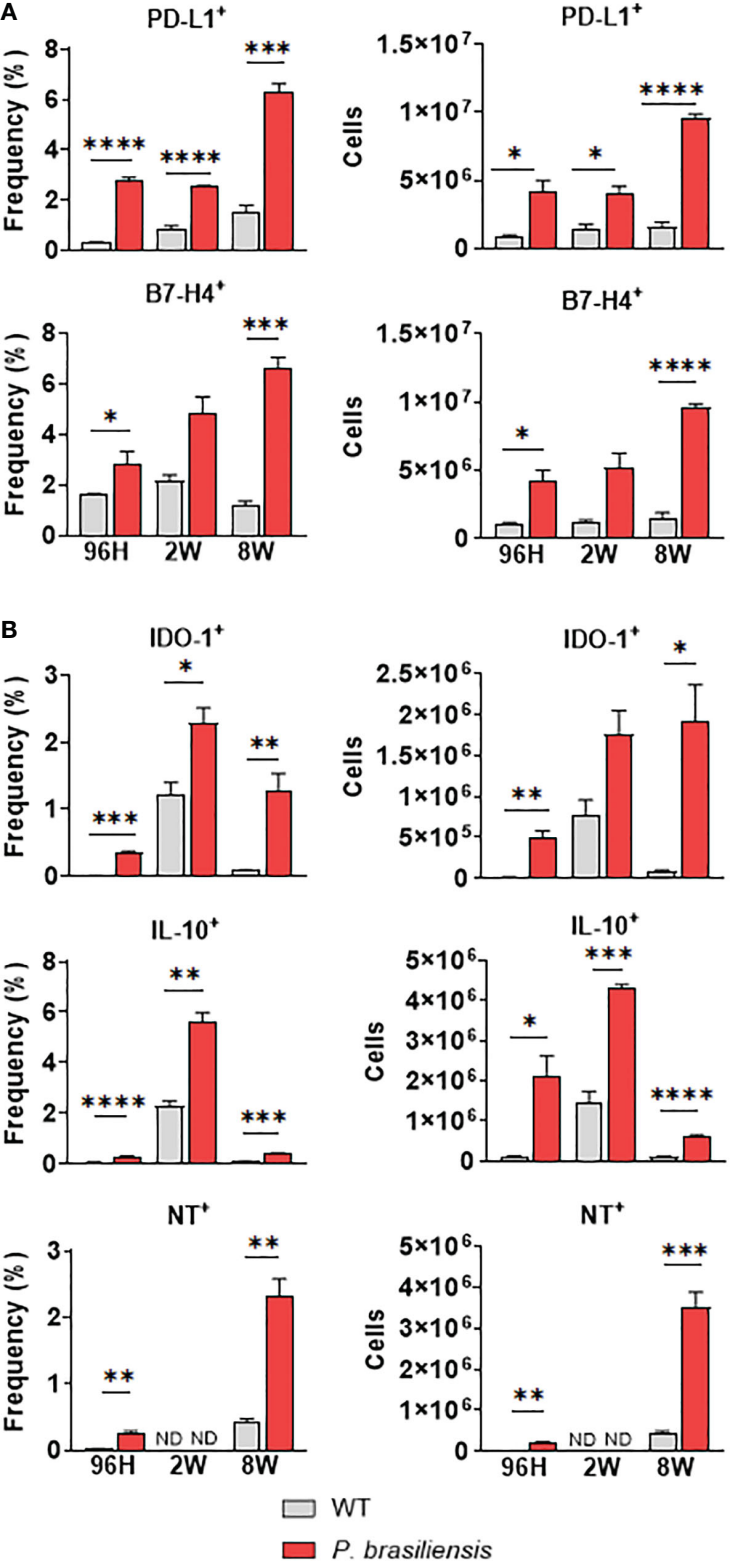
Immunosuppressive molecules expressed by polymorphonuclear-like MDSCs (PMN-MDSCs) in response to *P. brasiliensis* infection. The immunosuppressive molecules expressed by PMN-MDSCs were evaluated in the lung infiltrating cells of *P. brasiliensis*-infected mice (1×10^6^ yeasts cells) by flow cytometric analysis with 96 hours, 2 weeks, and 8 weeks of infection. The expression of cell membrane molecules such as **(A)** CD274 (PD-L1) and B7-H4 as well as intracellular molecules **(B)** IDO-1, IL-10, and nitrotyrosine (NT) were evaluated in CD45^+^CD11b^+^Ly6G^+^LY6C^low^ cells (PMN-MDSC). Cells were gated by FSC/SSC analysis. Data represent three independent experiments with 3-4 mice each. For comparisons between two groups, the mean ± SEM was obtained and analyzed by the unpaired Student’s *t*-test. Differences were considered significant when: *p<0.05; **p<0.01; ***p<0.001 and ****p<0.0001.

B7-H4 is also known to interact with T-lymphocytes, promoting their suppression through mechanisms that are as yet unknown (56). We detected low levels of both immunosuppressive molecules (B7-H4 and PD-L1) in PMN-MDSCs isolated from uninfected animals. Interestingly, we found that infection with *P. brasiliensis* promoted a massive influx of B7-H4^+^ and PD-L1^+^ PMN-MDSCs into the lungs. Furthermore, we detected a higher number of MDSCs expressing immunosuppressive molecules during the chronic phase of the disease (8 weeks of infection) compared with that during the acute phase of infection (**Figure 2A**).

IDO-1 promotes fungal clearance and inhibits T-cell immunity and inflammation, exhibiting its importance in host susceptibility to fungal infections (25, 57). Importantly, IDO-1 is expressed in several immune cells, including MDSCs (58, 59). In a previous study on pulmonary paracoccidioidomycosis, we demonstrated that the lack of IDO-1 expression caused an increased influx of activated Th17 cells to the lungs, with a simultaneous reduction in the number of Th1 and Treg cells (18). Therefore, we evaluated the production of this enzyme in MDSCs during *P. brasiliensis* infection. As depicted in **Figure 2B**, *P. brasiliensis* infection promoted an influx of PMN-MDSCs expressing IDO-1 at all the points studied. Interestingly, we observed highest frequencies of this MDSC subset in the second week of infection. However, regarding the absolute numbers, we again observed higher numbers in the eighth week of infection.

IL-10 has numerous suppressive functions associated with dampening of the immune inflammatory responses, including inhibition of myeloid cell maturation and interference in the development and function of T-cell phenotypes (60). Several studies have suggested that MDSCs are the predominant IL-10-producing cells in tumors and infections (61, 62). Here, we noticed that the expression of IL-10 by PMN-MDSCs was increased during all postinfection periods (**Figure 2B**). However, we observed a peak in the frequency and number of IL-10-expressing MDSCs in the second week of infection.

The inhibitory function of MDSCs has also been linked to the inducible nitric oxide synthase (iNOS) pathway, leading to nitric oxide (NO) production and reactive oxygen species (ROS) generation (63). As the interaction between NO and superoxide generates peroxynitrite, the presence of peroxynitrite-producing MDSCs can be assessed using an anti-nitrotyrosine antibody that detects nitrotyrosine (NT) residues present in intracellular compartments (64). We accordingly observed a slight increase in PMN-MDSCs expressing NT after 96 h of infection (**Figure 2B**). However, although we did not detect PMN-MDSC-NT^+^ at week 2, we observed higher frequencies and numbers of PMN-MDSC-NT^+^ at 8 weeks of infection.

### Expression of immunosuppressive molecules by M-MDSCs during *P. brasiliensis* infection

**Figure 3:**
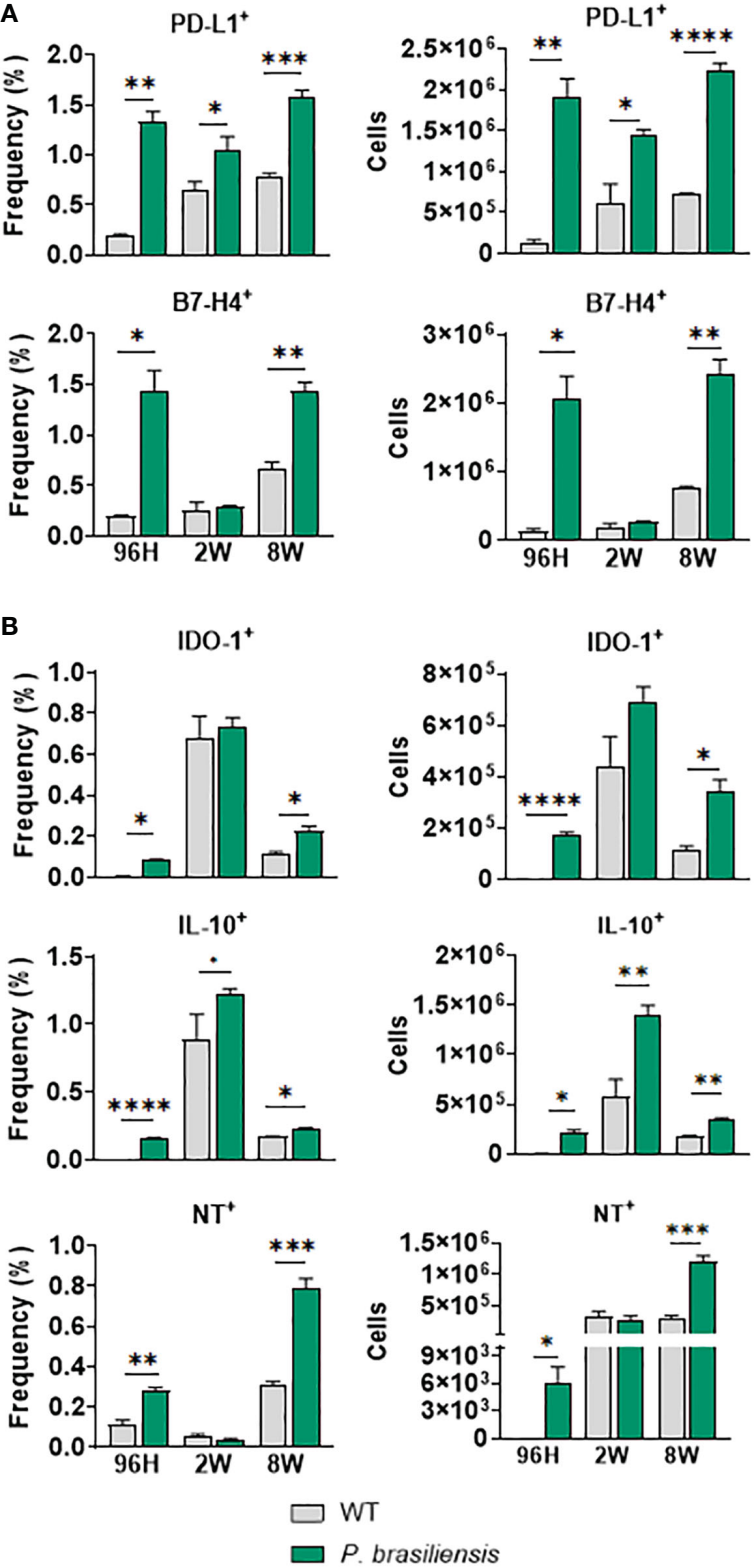
Immunosuppressive molecules expressed by Monocytic-like-MDSCs (M-MDSCs) in response to *P. brasiliensis* infection. The expression of immunosuppressive molecules by M-MDSC was evaluated in the lung infiltrating cells of *P. brasiliensis*-infected mice (1×10^6^ yeasts cells) by flow cytometric analysis with 96 hours, 2 weeks, and 8 weeks of infection. The expression of cell membrane molecules **(A)** such as CD274 (PD-L1) and B7-H4, as well as intracellular molecules **(B)** IDO-1, IL-10, and nitrotyrosine (NT), were evaluated in CD45^+^CD11b^+^LY6G^-^LY6C^hi^ cells (M-MDSC). Cells were gated by FSC/SSC analysis. Data represent three independent experiments with 3-4 mice each. For comparisons between two groups, the mean ± SEM was obtained and analyzed by the unpaired Student’s *t*-test. Differences were considered significant when: *p<0.05; **p<0.01; ***p<0.001 and ****p<0.0001.

Similar to the PMN-MDSC subset, M-MDSCs expressing immunosuppressive molecules, such as B7-H4 and PD-L1, were also present in greater frequency and numbers in lung-infiltrating leukocytes of *P. brasiliensis*-infected mice than in uninfected animals after 96 h and 8 weeks of infection. However, we did not observe any differences in the expression of B7-H4 at week 2 of infection (**Figure 3A**). Moreover, we found an increased presence of M-MDSCs expressing IDO-1, IL-10, and NT in almost all infection periods, except for IDO-1^+^ and NT^+^ M-MDSCs at week 2 of infection, for which no differences were detected (**Figure 3B**).

### *In vitro*-generated BM-MDSCs and Lung-MDSCs suppressed T-cell responses

**Figure 4:**
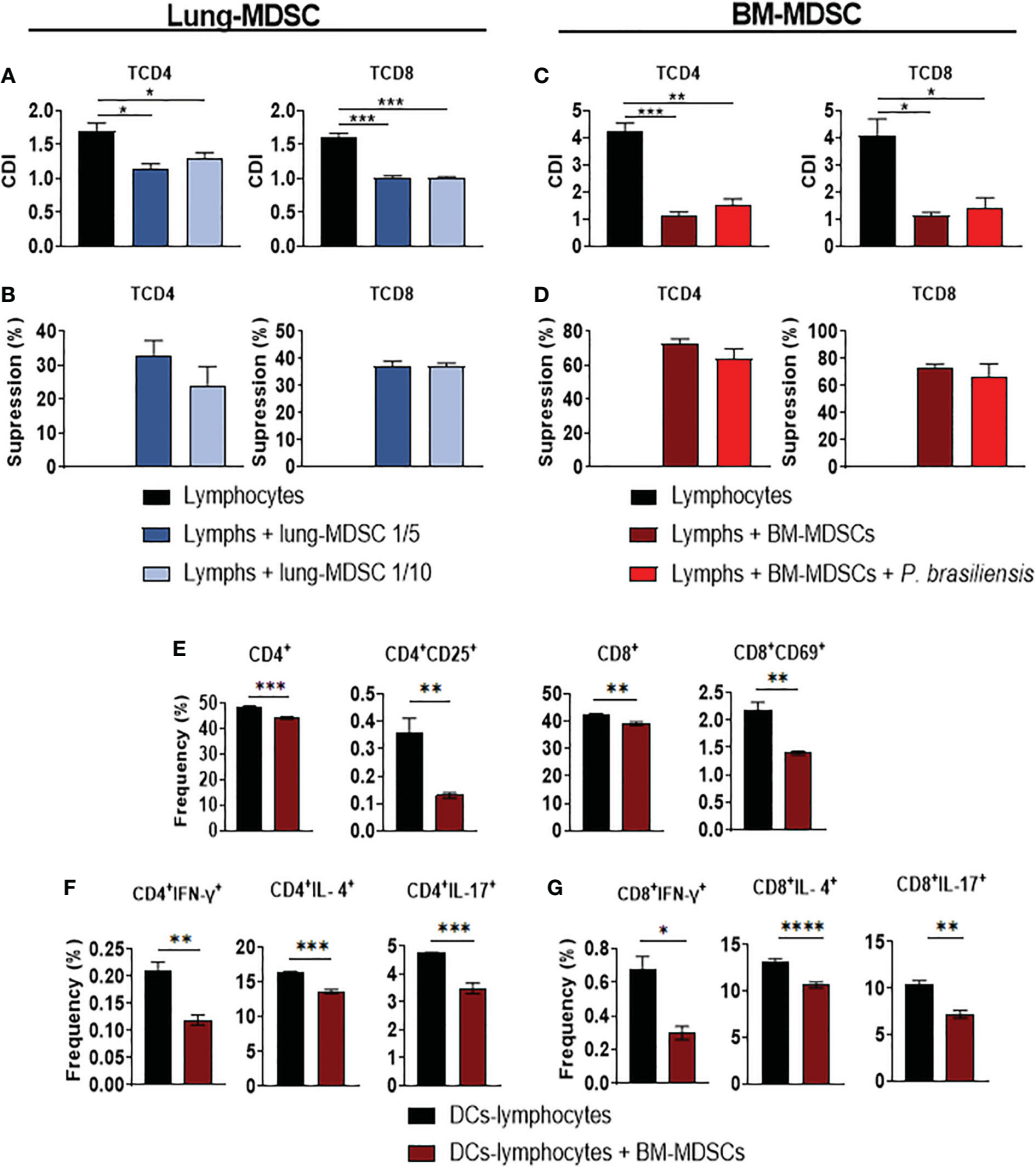
Suppression of T-cell responses by MDSCs. To evaluate the ability of MDSCs to suppress T cell proliferation, MDSCs were obtained from the lungs of *P. brasiliensis-*infected mice after two weeks of infection (Lung-MDSCs). Alternatively, bone marrow-derived MDSCs (BM-MDSCs) were generated *in vitro* and challenged overnight or not by *P. brasiliensis* at a ratio of 1:50 (yeast: MDSCs), as indicated in the material and methods. Both Lung-MDSCs **(A, B)** and BM-MDSCs **(C, D)** were sorted and subsequently cocultured with CFSE-labeled CD3^+^ T cells, previously activated with anti-CD3/CD28 antibodies. Following coculture for four days (ratios of 1:10 and 1:5 -MDSC: lymphocytes- for L-MDSCs and ratio of 1:10 for BM-MDSCs), the proliferation of the CD4 and CD8 T cells was characterized by flow cytometric analysis, and the cell division index (CDI) was obtained **(A, C)**. To better evaluate the ability of Lung-MDSCs **(B)** and BM-MDSCs **(D)** to suppress lymphoproliferation, the percentage of T cell suppression was calculated from CDI results. To evaluate the ability of BM-MDSCs to suppress activated T cells **(E)** and Th/Tc lymphocytes differentiation **(F, G)**, spleen lymphocytes from *naïve* mice were cocultured with BM-DCs that were previously challenged with *P. brasiliensis* yeasts (ratio 1:50; yeast-DCs). After 24 hours of DC-Lymphocyte coculture, the BM-MDSCs were added to the coculture (ratio of 1:10; MDSC: T cells) and T cell responses were compared to cocultures without MDSCs. After four days of coculture, lymphocytes were evaluated by flow cytometry for total and activated T cells (CD4^+^, CD4^+^CD25^+^, CD8^+^ and CD8^+^CD69^+^) **(E)**, and for intracellular expression of Th-signature cytokines by CD4+ T cells (Th1: CD4^+^IFN-γ^+^; Th2: CD4^+^IL-4^+^, Th17: CD4^+^IL-17^+^) **(F)**, and CD8+ T signature cytokines (Tc1: CD8^+^IFN-γ^+^, Tc2: CD8^+^IL-4^+^, Tc17: CD8^+^IL-17^+^ **(G)**. Data represent two independent experiments. For comparisons between two groups, the mean ± SEM was obtained and analyzed by the unpaired Student’s *t*-test. Differences were considered significant when: *p<0.05; **p<0.01; ***p<0.001 and ****p<0.0001.

The ability of MDSCs to suppress T-cell responses has been demonstrated in tumor and infectious pathologies (29, 65). To investigate the effects of MDSCs on T-cells, we sorted and cocultured *in vitro*- and lung-isolated MDSCs with CFSE-labeled CD3^+^ T–cells for 4 d. We assessed the proliferation of CD4^+^ and CD8^+^ T-cells using flow cytometry. As depicted in **Figures 4A–D**), the proliferation of CD4^+^ and CD8^+^ T-cells was inhibited by both cell preparations; however, higher suppressive activity was observed when BM-MDSCs were used (**Figures 4C, D**).

Next, we examined the capacity of BM-MDSCs to inhibit the differentiation of T helper (Th) subsets. We cocultured spleen lymphocytes isolated from *naïve* mice with BM-DCs previously challenged with *P. brasiliensis*. After 24 h of coculture with DC and lymphocytes, BM-MDSCs were added to the culture. After 4 d, we performed an intracellular flow cytometric assay to detect the proliferation of CD4^+^ and CD8^+^ T-cells and of those secreting IFN-γ, IL-4, and IL-17. We specifically found that BM-MDSCs inhibited the proliferation and activation of CD4^+^ and CD8^+^ T-cells (**Figure 4E**). We also detected that the frequency of CD4^+^ T-cells expressing either IFN-γ, IL-4, or IL-17 was decreased in the cocultures with BM-MDSCs (**Figure 4F**). Moreover, the frequency of CD8^+^ T-cells expressing either IFN-γ, IL-17 and IL-4 was decreased in the presence of BM-MDSCs (**Figure 4G**). Taken together, our results demonstrated that *P. brasiliensis* infection induced an influx of MDSCs in the lungs, thus exerting a significant suppressive effect on the proliferation and activation of CD4^+^ and CD8^+^ T-cells.

### Depletion of Gr1^+^ cells in *P. brasiliensis-*infected mice promoted a robust decrease in PMN-MDSCs, but not M-MDSCs

**Figure 5:**
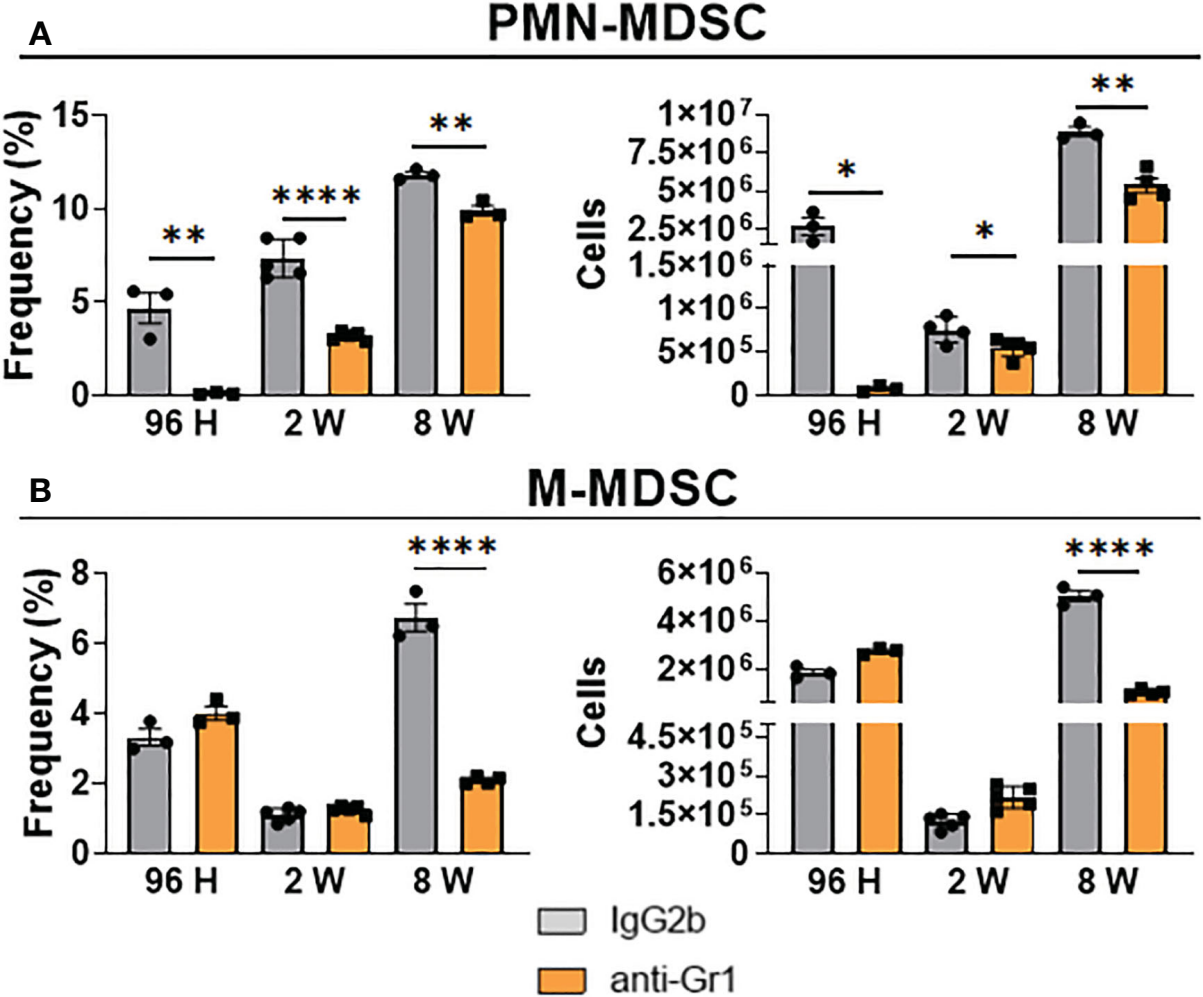
Effect of MDSCs depletion by anti-Gr1 treatment in the presence of pulmonary PMN-MDSCs and M-MDSCs at different periods of *P. brasiliensis* infection. C57BL/6 WT mice were infected i.t. with 1×10^6^ yeasts cells of *P. brasiliensis*. After 24 hours, 1 week, and 7 weeks of infection a group of mice received intraperitoneal injections of anti-Gr1 antibody (clone RB6-8C5) or rat IgG2b isotype control antibody (200 µg/dose). After 96 hours, 2 weeks, and 8 weeks of infection lungs were removed, leukocytes were obtained and the frequency and number of PMN-MDSCs **(A)** and M-MDSCs **(B)** subsets were analyzed by flow cytometry. Data represent 3 independent experiments with 3-4 mice each. For comparisons between two groups, the mean ± SEM was obtained and analyzed by the unpaired Student’s *t*-test. Differences were considered significant when: *p<0.05; **p<0.01 and ****p<0.0001.

**Figure 6:**
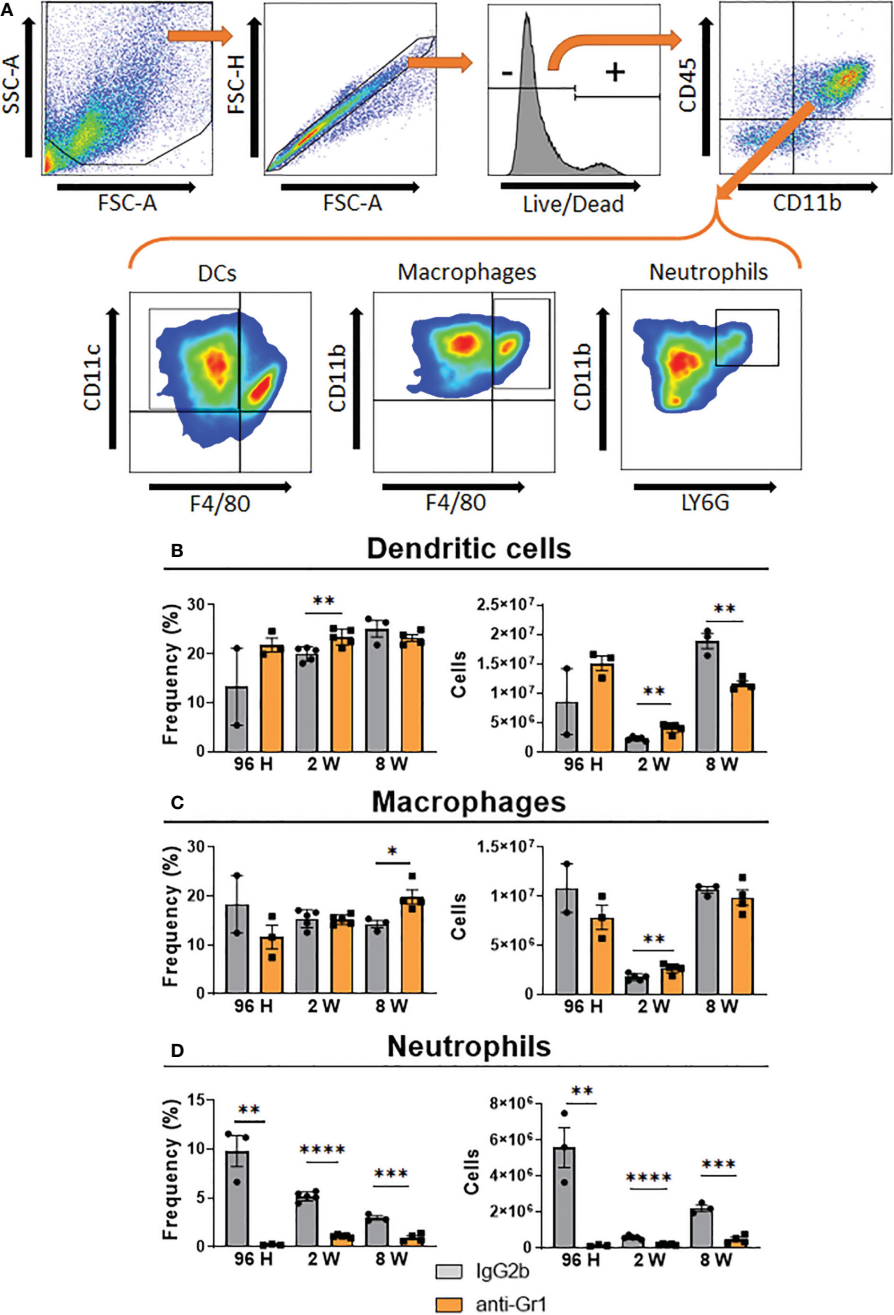
Effect of MDSC depletion in the presence of pulmonary myeloid cells of *P. brasiliensis*-infected mice. The frequency and the absolute number of pulmonary myeloid cells were evaluated in the lung-infiltrating leukocytes of anti-Gr1-treated and untreated infected mice. After 24 hours, 1 week, and 7 weeks of infection a group of mice received intraperitoneal injections of anti-Gr1 antibody (clone RB6-8C5) or rat IgG2b isotype control antibody (200 µg/dose). At 96 hours, 2 weeks, and 8 weeks of infection lungs were removed and leukocytes were obtained. The cells were gated by FSC/SSC analysis **(A)** and dendritic cells CD45^+^CD11b^+^CD11c^+^F4/80^-^ **(B)**, macrophages CD45^+^CD11b^+^F4/80^+^ **(C)** and neutrophils CD45^+^CD11b^+^Ly6G^hi^ **(D)** were analyzed by flow cytometry. Data represent three independent experiments with 3-4 mice each. For comparisons between two groups, the mean ± SEM was obtained and analyzed by the unpaired Student’s *t*-test. Differences were considered significant when: *p<0.05; **p<0.01; ***p<0.001 and ****p<0.0001.

We used an anti-Gr1 (clone RB6-8C5) antibody, which has been employed to deplete MDSCs in mice (34, 66), to deplete these cells in the pulmonary PCM model. We employed three protocols (described in Materials and Methods) to deplete MDSCs at different periods of *P. brasiliensis* infection, allowing the evaluation of their activity during the acute (96 h), intermediate (2 weeks), and chronic phase of infection. Expectedly, we found that both the frequency and number of PMN-MDSCs were dramatically decreased after 96 h, 2 weeks, and 8 weeks of infection (**Figure 5A**). We specifically observed a significant reduction in the frequency and number of M-MDSCs only at week 8 post-infection (**Figure 5B**). Unexpectedly, we did not detect any differences in the frequency or number of M-MDSCs at 96 h and 2 weeks after infection. This unexpected finding was similar to those obtained when evaluating the effect of anti-Gr1 treatment on the presence of other myeloid cells (DCs, macrophages, and neutrophils) in the lungs of infected mice. As shown in **Figure 6**, anti-Gr1 treatment had a slight but significant effect on the increase in the frequency and number of DCs after 2 weeks of infection; however, the longer infection time resulted in a lower number of DCs (**Figure 1B**). Similarly, we observed an increase in the frequency of macrophages at 8 weeks of infection as well as a higher number of macrophages at the second week post-infection in mice treated with anti-Gr1 (**Figure 6C**). Macrophages were not negatively affected by anti-Gr1 treatment; instead, a robust suppressive effect on the presence of neutrophils (**Figure 6D**) migrating to the lungs of *P. brasiliensis-*infected mice was observed.

### Depletion of Gr1^+^ cells increased the number of CD4^+^ T-cells but decreased the presence of CD8^+^ T-lymphocytes in the lungs of *P. brasiliensis-*infected mice

**Figure 7:**
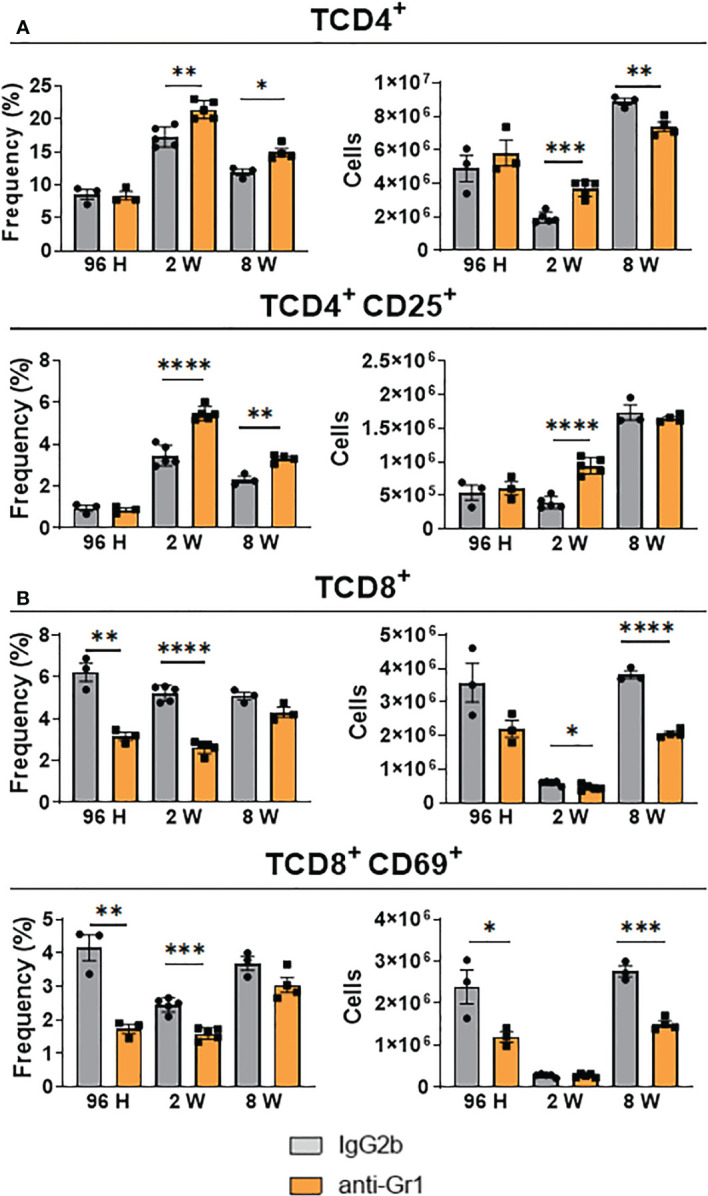
Effect of MDSC depletion in the presence of pulmonary T lymphocytes of *P. brasiliensis*-infected mice. The frequency and the absolute number of total and activated CD4 **(A)** and CD8 **(B)** T cells were evaluated in the lung-infiltrating leukocytes of anti-Gr1-treated and untreated infected mice. After 24 hours, 1 week, and 7 weeks of infection, a group of mice received intraperitoneal injections of anti-Gr1 antibody (clone RB6-8C5) or rat IgG2b isotype control antibody (200 µg/dose). After 96 hours, 2 weeks, and 8 weeks of infection lungs were removed, and leukocytes were obtained. The cells were gated by FSC/SSC analysis and lymphocytes were gated for CD4, CD25, CD8, and CD69 expression. Data represent three independent experiments with 3-4 mice each. For comparisons between two groups, the mean ± SEM was obtained and analyzed by the unpaired Student’s *t*-test. Differences were considered significant when: *p<0.05; **p<0.01; ***p<0.001 and ****p<0.0001.

As previously described, anti-Gr1 treatment modified the composition of myeloid cell subsets present in the lungs of *P. brasiliensis-*infected mice, indicating that this diverse range of cells might affect T-cell immunity against this fungal pathogen. Therefore, following the depletion of MDSCs, we evaluated the influx and activation of CD4^+^ and CD8^+^ T-cells. We found that the frequency and absolute number of CD4^+^ and CD4^+^ CD25^+^ T-cells did not change following anti-Gr1 treatment during the acute phase (96 h) of infection. However, the frequency and number of CD4^+^ and CD4^+^ CD25^+^ T-cells were significantly higher in the lungs of anti-Gr1-treated mice than in the control mice after 2 and 8 weeks of infection (**Figure 7A**). Surprisingly, we obtained the opposite results when the CD8^+^ T-cell population was examined. We detected a decreased frequency and number of CD8^+^ and CD8^+^ CD69^+^ T-lymphocytes during all phases of the disease (**Figure 7B**).

### Anti-Gr1 treatment in *P. brasiliensis*-infected mice enhanced Th1 and Th17 responses

**Figure 8:**
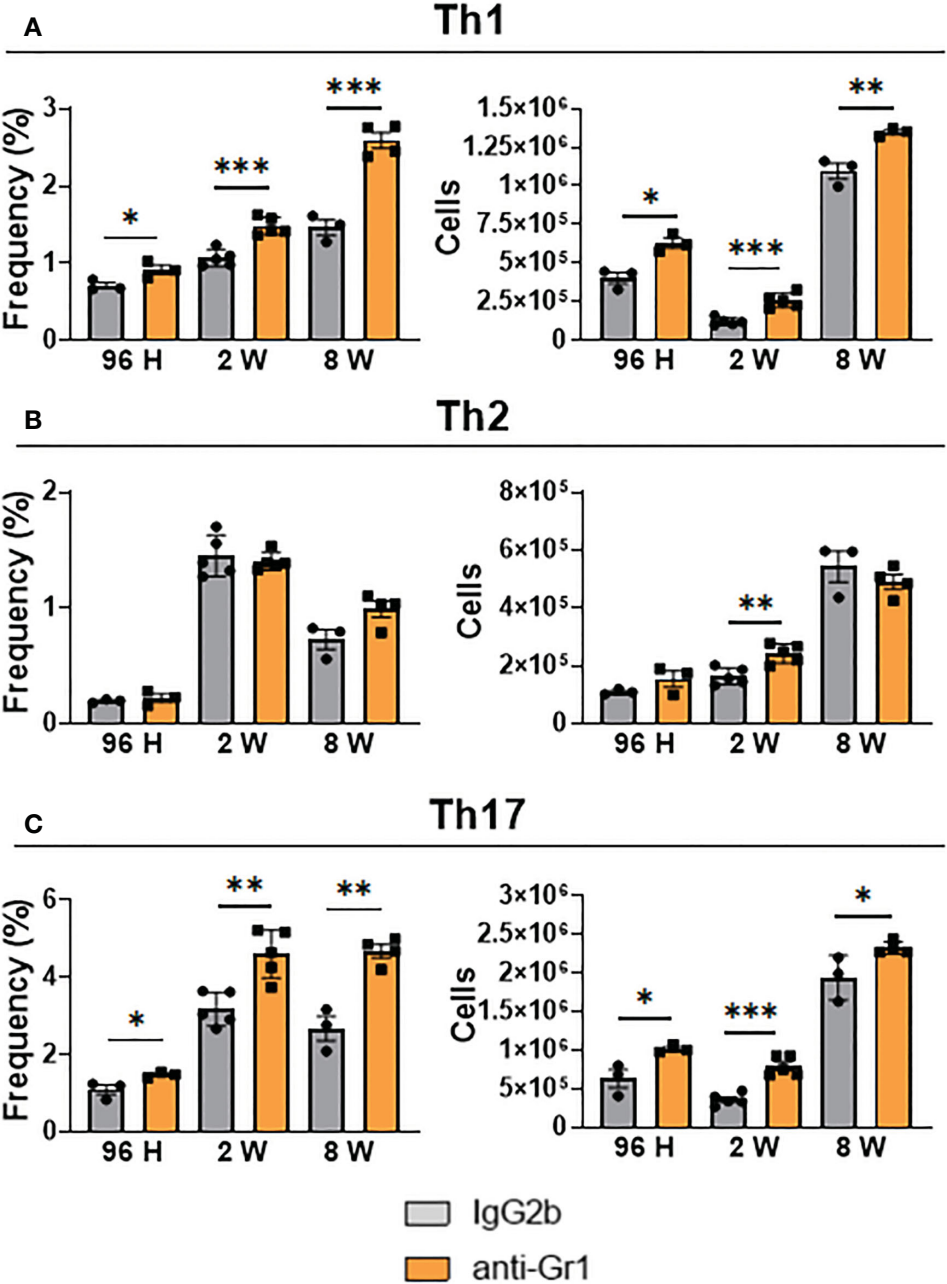
Effect MDSC depletion in the presence of pulmonary Th1, Th2, and Th17 lymphocytes of *P. brasiliensis*-infected mice. The presence of intracellular IFN-γ, IL-4, and IL-17 in CD4^+^ T cells in the lung infiltrating leukocytes was assessed by intracellular cytokine staining by flow cytometry. After 24 hours, 1 week, and 7 weeks of infection a group of mice received intraperitoneal injections of anti-Gr1 (clone RB6-8C5) or rat IgG2b isotype control antibody (200 µg/dose). After 96 hours, 2 weeks, and 8 weeks of infection, lung cells were stimulated *in vitro* with PMA/ionomycin + brefeldin A for 6 hours and subjected to intracellular staining. The lung-infiltrating lymphocytes were gated by FSC/SSC analysis. Lymphocytes were gated for CD4 expression and then for IFN-γ **(A)**, IL-4 **(B)**, and IL-17 **(C)** expression. Results are expressed in frequency and the absolute number of cells and are representative of three independent experiments. For comparisons between two groups, the mean ± SEM was obtained and analyzed by the unpaired Student’s *t*-test. Differences were considered significant when: *p<0.05; **p<0.01 and ***p<0.001.

We also measured the frequency and absolute number of Th1, Th2, and Th17 cells in the lungs of anti-Gr1-treated and untreated mice using flow cytometry. We found that the frequency and number of CD4^+^ T-cells expressing intracellular IFN-γ^+^ (Th1 cells) were significantly higher in the lungs of anti-Gr1-treated mice than in untreated mice (**Figure 8A**). We obtained similar results when evaluating the presence of CD4^+^ IL-17^+^ (Th17) cells (**Figure 8C**). In contrast, depletion of Gr1^+^ cells did not affect the frequency of CD4^+^ IL-4^+^ (Th2) cells but significantly increased this T-cell subset at week 2 of infection (**Figure 8B**).

### Anti-Gr1 treatment enhanced the secretion of Th1 and Th17 cytokines after 2 weeks of infection but reduced the levels of Th1-, Th2-, and Th17-secreted cytokines in the chronic phase

**Figure 9:**
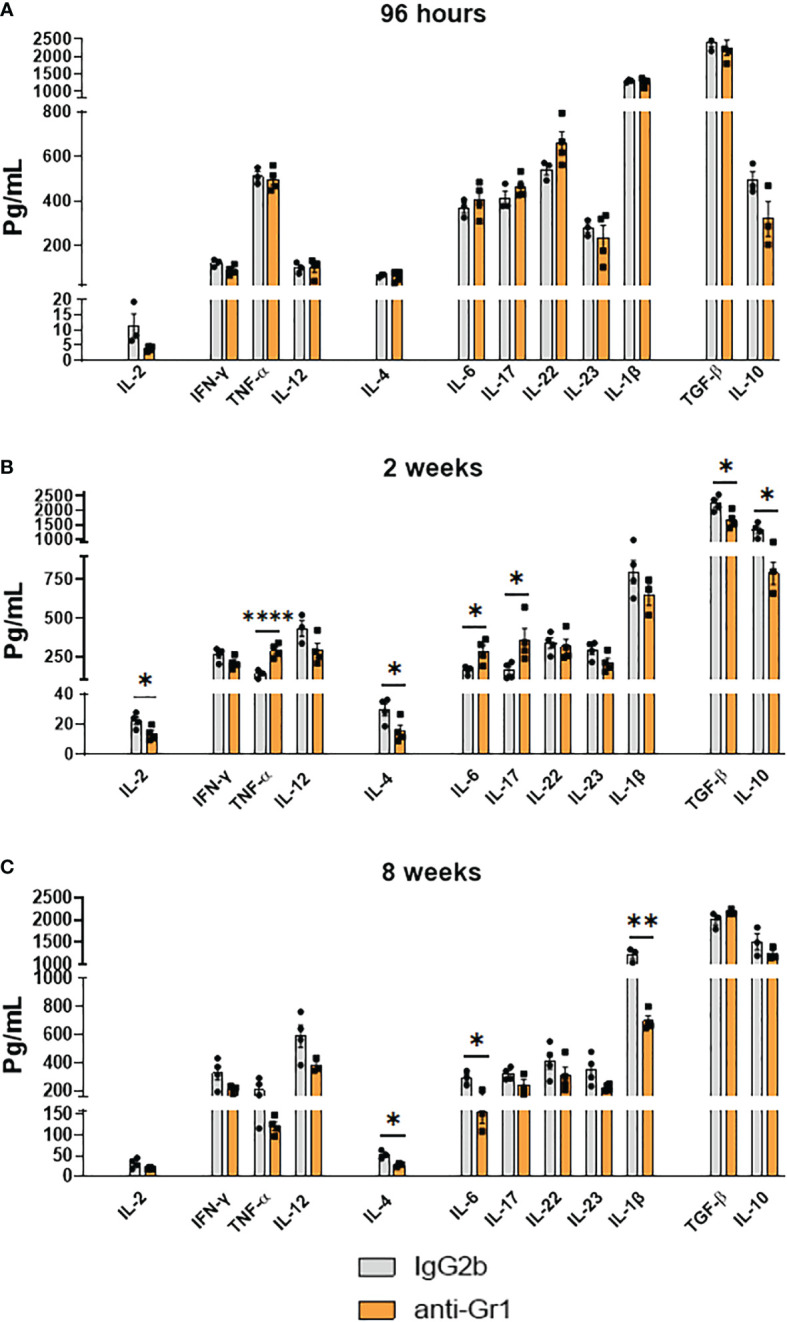
Effect of MDSC depletion in the levels of pulmonary cytokines of *P. brasiliensis* infected mice. Levels of cytokines in organ homogenates of anti-Gr1 and IgG2b control-treated mice were measured after i.t. infection with 1 × 10^6^ yeast cells. Lungs were collected, disrupted in 5.0 ml of PBS, and the supernatants obtained were analyzed for cytokine content by capture ELISA. The levels of IL-2, IFN-γ, TNF-α, IL-12, IL-4, IL-6, IL-17, IL-22, IL-23, TGF-β, IL-1β, and IL-10 were analyzed at 96 hours **(A)**, 2 weeks **(B)**, and 8 weeks **(C)** of infection. Data represent 3 independent experiments with 3-4 mice each. For comparisons between two groups, the mean ± SEM was obtained and analyzed by the unpaired Student’s *t*-test. Differences were considered significant when: *p<0.05; **p<0.01 and ****p<0.0001.

We measured the levels of type 1 (IFN-γ, TNF-α, IL-12, and IL-2), type 2 (IL-4), Th17-associated (IL-6, IL-22, IL-23, and IL-17), and MDSC-produced cytokines (IL-1β, IL-10, and TGF-β) in the lungs of anti-Gr1-treated mice and their IgG2b-treated control counterparts at 96 h, 2 weeks, and 8 weeks after *P. brasiliensis* infection. No differences were detected in the lung homogenates between the 2 groups after 96 h of infection (**Figure 9A**). However, we noticed that the lungs of Gr1-depleted mice showed augmented levels of cytokines related to Th1 (TNF-α) and Th17 (IL-6 and IL-17) profiles at 2 weeks after infection. Furthermore, corroborating with the depletion of MDSCs, IL-10 and TGF-β levels were diminished in the lungs of anti-Gr1-treated mice compared with those in IgG2b-treated control mice. We observed lower levels of IL-4 related to Th2 cells and IL-2, which are important for T-cell activation and Treg cell expansion, in the lung homogenates of anti-Gr1-treated mice compared with those in IgG2b-treated controls (**Figure 9B**). However, we did not detect any differences in the production of IFN-y, IL-12, IL-22, IL-23, or IL-1β between the control and anti-Gr1 groups after 2 weeks of infection. The levels of Th2 cytokine (IL-4) remained low in the lungs of anti-Gr1-treated mice at 8 weeks after infection. Furthermore, we noticed that the levels of IL-1β, a cytokine related to the induction and maintenance of MDSCs in the context of fungal infection (32), were reduced after anti-Gr1 treatment. We obtained similar results on evaluating the levels of IL-6. Finally, we detected equivalent levels of IL-2, IFN-y, TNF-α, IL-12, IL-17, IL-22, IL-23, TGF-β, and IL-10 in the lungs of anti-Gr1-treated and control mice during the chronic phase of infection (**Figure 9C**).

### Anti-Gr1 treatment protected mice from high fungal loads and lung pathology during the chronic phase of infection

**Figure 10:**
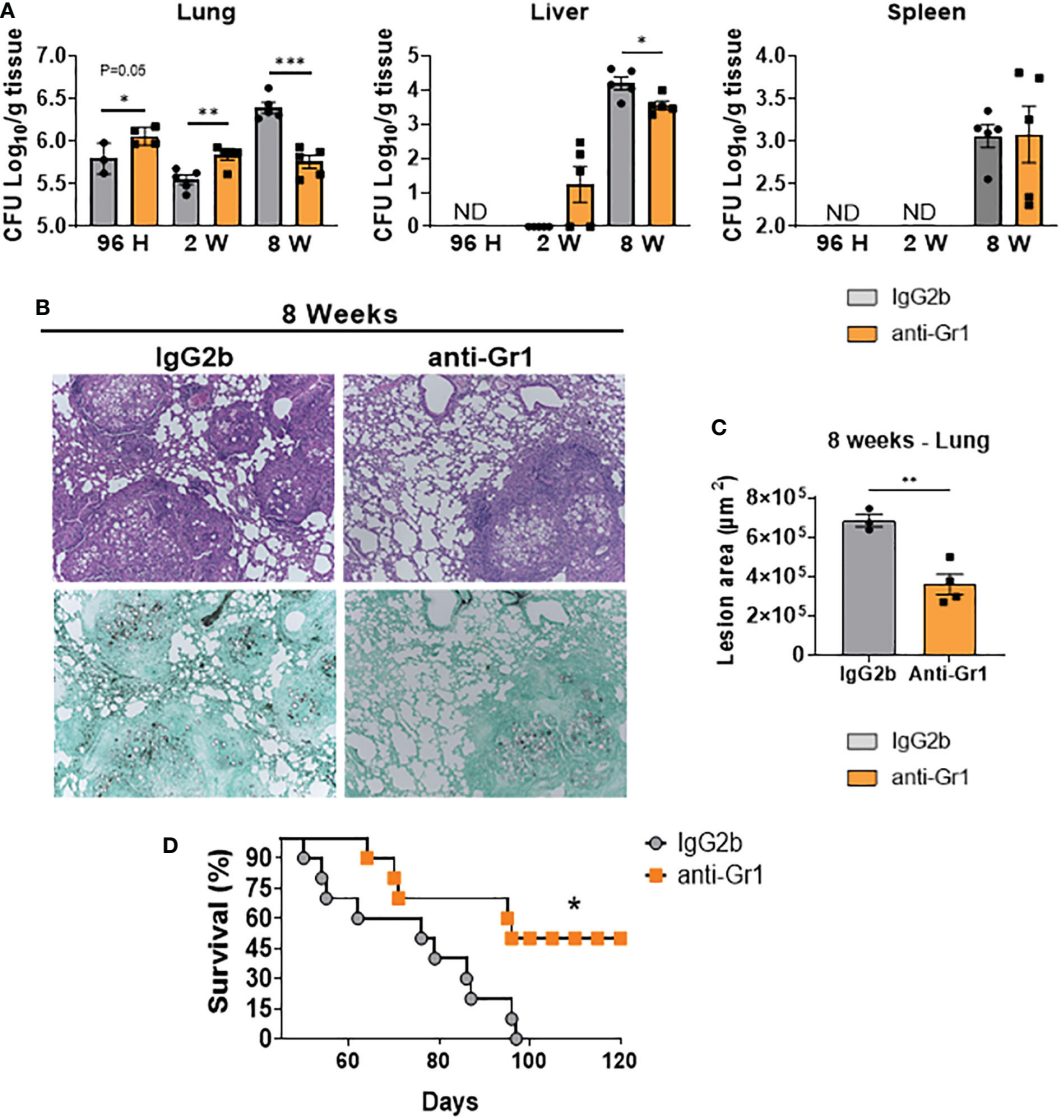
Depletion of MDSCs reduces fungal loads and tissue injury at the chronic phase of the disease and reduces mortality rates of *P. brasiliensis*-infected mice. C57BL/6 WT mice were infected i.t. with 1×10^6^ yeasts cells of *P. brasiliensis*. After 24 hours, 1 week, and 7 weeks of infection a group of mice received intraperitoneal injections of anti-Gr1 (clone RB6-8C5) or rat IgG2b isotype (200 µg/dose). Colony-forming unit (CFU) counts from lungs, liver, and spleen were determined 96 hours, 2 weeks, and 8 weeks after *P. brasiliensis* infection **(A)**. Photomicrographs of lung lesions of control and anti-Gr1-depleted mice at week 8 of infection. Lesions were stained with hematoxylin-eosin (top panels) and Grocott (bottom panels) **(B)**. Total area of lesions in the lungs at 8 weeks of infection. **(C)**. Survival curves of MDSCs-depleted and control infected mice were determined for a period of 120 days **(D)**. The bars represent means ± standard errors of the mean (SEM) of log10 CFU counts obtained from groups of 3–4 mice. For comparisons between two groups, the mean ± SEM was obtained and analyzed by the unpaired Student’s *t*-test. Differences were considered significant when: *p<0.05; **p<0.01 and ***p<0.001.

To verify whether depletion of MDSCs affects disease severity, we examined the target organs of anti-Gr1-treated and control mice using CFU counts and lung histopathology. We detected higher fungal loads in the lungs of anti-Gr1-treated mice after 96 h and 2 weeks of infection compared with those in the control group (**Figure 10A**). However, we did not observe any dissemination to the liver and spleen after 96 h but detected a small number of viable fungal cells at week 2 post-infection in the liver of anti-Gr1-treated mice. In contrast, we obtained the opposite results during the chronic phase of infection. Compared with the IgG2b control mice, anti-Gr1-treated mice had reduced pulmonary and hepatic fungal burdens. We also observed fungal dissemination to the spleen at week 8 after infection but did not detect any differences between the treatment and control groups. We also analyzed histological sections of the lungs from anti-Gr1- or IgG2b-treated mice to characterize inflammatory reactions (HE staining) and the presence of fungal cells (Grocott, methamine silver staining). As shown in **Figure 10B**, at week 8 of infection, anti-Gr1-treated mice showed a reduced pattern of inflammatory reactions and diminished presence of fungal cells inside the lesions. We measured the lesion areas in the lungs of both groups and observed a significant reduction in anti-Gr1-treated mice (**Figure 10C**). Importantly, we found that the reduced fungal burden and tissue pathology resulted in reduced mortality in anti-Gr1-treated mice (**Figure 10D**). These data led us to conclude that the reduction in the number of MDSCs in ongoing PCM exerts a protective effect on infected hosts.

### Adoptive transfer of pulmonary MDSCs aggravated disease in *P. brasiliensis*-infected mice

**Figure 11:**
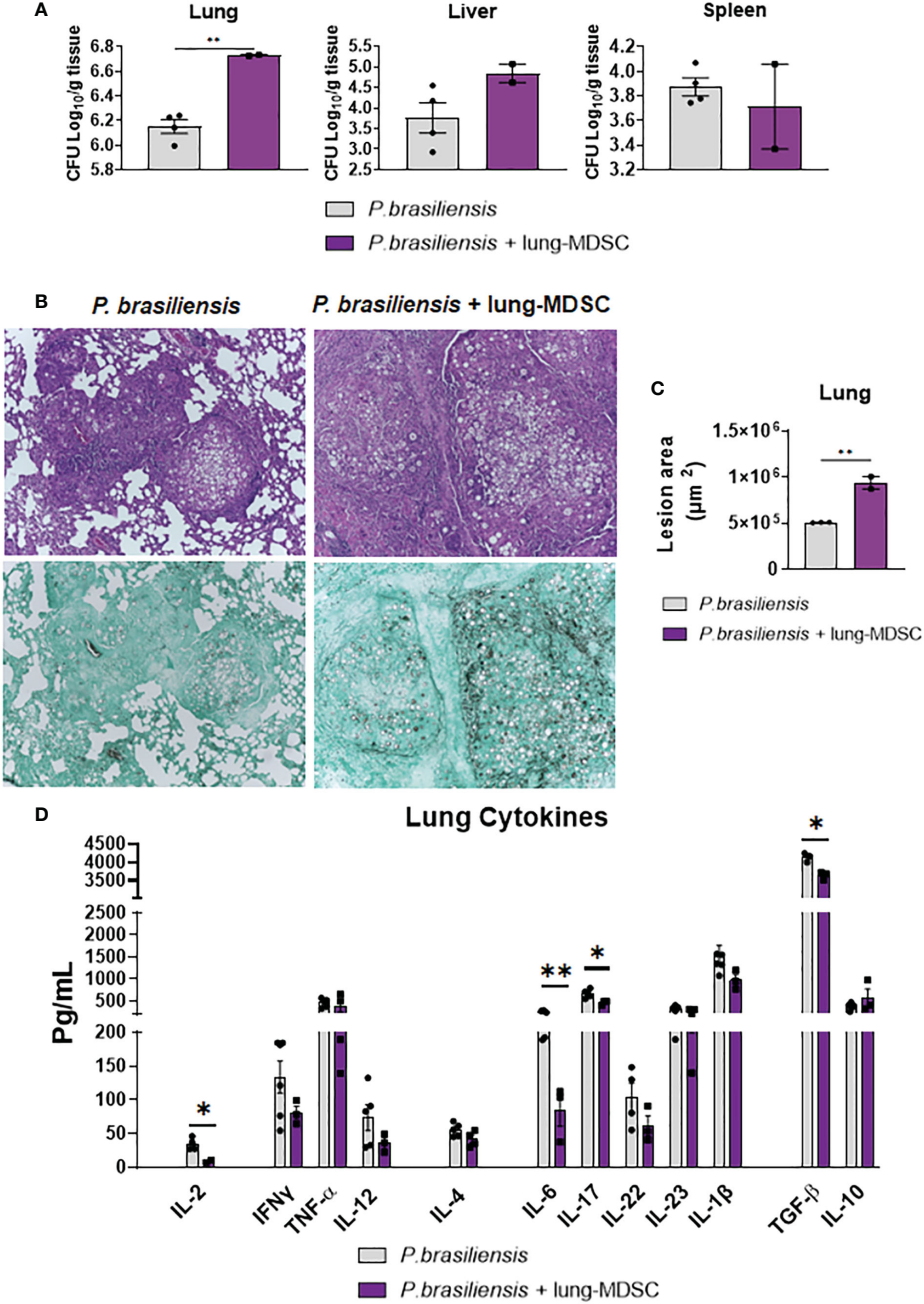
Adoptive transfer of MDSCs to *P. brasiliensis*-infected mice increases disease severity. C57BL6/WT mice were infected with *P. brasiliensis*; after 2 weeks, the lungs were obtained and MDSCs were isolated. The cells were transferred into recipient mice that had been infected with *P. brasiliensis* 7 weeks before the transfer. Infected control animals received only DMEN medium. Seven days after the adoptive transfer of Lung-MDSCs, disease severity was measured by CFU counts **(A)**. Photomicrographs of lung lesions of control and MDSC-recipient mice at week 8 of infection. Lesions were stained with hematoxylin-eosin (top panels) and Grocott (bottom panels) **(B)**. The total area of lung lesions was calculated in square micrometers of 5 microscopic fields per slide in 3-4 mice per group **(C)**. Pulmonary cytokines (IL-2, IFN-γ, TNF-α, IL-12, IL-4, IL-6, IL-17, IL-22, IL-23, TGF-β, IL-1β, and IL-10) were measured by ELISA in lung homogenates **(D)**. Data represent two independent experiments with 3-4 mice per group. For comparisons between two groups, the mean ± SEM was obtained and analyzed by the unpaired Student’s *t*-test. Differences were considered significant when: *p<0.05 and **p<0.01.

To better characterize the importance of MDSCs in pulmonary PCM, we employed a complementary approach wherein MDSCs were adoptively transferred to *P. brasiliensis-*infected mice. Briefly, we infected donor mice with *P. brasiliensis*, and 2 weeks later, obtained their lungs and MDSCs. We transferred MDSCs to recipient mice that had been infected with *P. brasiliensis* 7 weeks earlier. The adoptive MDSC transfer was performed 7 weeks after infection to allow the evaluation of the effect of the MDSC transfer to animals with established severe disease. Furthermore, the effect of the depletion of MDSCs is evident during the chronic phase. We evaluated disease severity 7 d after MDSCs transfer using CFU counts, lung histopathology and by measuring the production of cytokines. As indicated in **Figure 11**, adoptive transfer of MDSCs obtained from the lungs of *P. brasiliensis*-infected mice led to more severe disease. We found that the pulmonary fungal loads were higher in mice that received adoptive transfer of MDSCs than in those that received only DMEM (control; **Figure 11A**). Furthermore, we observed that the lung histopathological appearance of MDSC-recipient mice was considerably more severe than that of control mice. Particularly, lung sections from all MDSC-recipient mice showed numerous confluent granulomatous lesions containing a high number of yeast cells occupying large areas of the lung parenchyma. In contrast, lung sections from control mice revealed better preservation of the pulmonary tissue and fewer yeast cells (**Figure 11B**). Morphometric analyses of histological sections are shown in **Figure 11C**. We also noticed that the lesion areas were significantly larger in the lungs of MDSC-recipient mice than in the lungs of control mice. Importantly, these results were opposite to those obtained in experiments wherein MDSCs were partially depleted. As expected, in an immunosuppressive environment, the levels of several cytokines, such as IL-2, IL-6, TGF-β, and IL-17, were reduced in pulmonary homogenates from MDSC-recipient mice (**Figure 11D**).

## Discussion

Recently, MDSCs have been reported as protagonists in the pathogenesis of several chronic infections (31–34, 47). Nevertheless, the role of these cells in PCM remains unclear. Our study provided the first evidence of a deleterious effect of this cell population in the chronic phase of pulmonary PCM. We investigated the contribution of MDSCs to host defenses against *P. brasiliensis*. Following pulmonary infection in C57BL/6 WT mice, considerable recruitment of PMN- and M-MDSCs to the lungs was observed. Theoretically, these recruited MDSCs should suppress T-cell responses as both MDSC subsets secrete the immunoregulatory cytokine IL-10 and the IDO-1 enzyme, which exerts immunosuppressive activity. Moreover, the high expression of cell membrane molecules involved in T-cell suppression, that is, B7-H4 and PD-L1, indicated that the recruitment of MDSCs contributed to immunosuppression during *P. brasiliensis* infection. Therefore, the accumulation of both MDSC subsets in the lungs of infected mice, along with the expression of common immunosuppressive mechanisms, supported their involvement in disease progression. Further data obtained following depletion of MDSCs and MDSC-lymphocyte coculture also confirmed the ability of MDSCs to suppress T-cell responses in pulmonary PCM.

The suppressive effect of MDSCs on T-lymphocytes has been described in tumors (45), bacterial (34), viral (67), protozoal (68), and fungal infections (38, 47). Based on this, we used two protocols to evaluate the expansion of T-lymphocytes in contact with suppressive cells. Both MDSC populations, that is, those obtained from the pulmonary inflammatory focus (lung-MDSCs) and those derived from normal bone marrow cells (BM-MDSCs) *in vitro* were able to impair the proliferation of CD4^+^ and CD8^+^ T-lymphocytes. These data were in agreement with the aforementioned studies on lymphocyte suppression. Interestingly, the presence of fungi in the coculture did not interfere with the ability of MDSCs to suppress the activation of lymphocytes *in vitro*, indicating that the interaction with fungal cells did not increase the immunosuppressive ability of MDSCs.

To address the role of MDSCs in different disease scenarios, several strategies, including the use of anti-Gr1 antibodies and pharmacological approaches, have been widely used in mouse models to selectively eliminate MDSCs (34, 46, 47, 69). However, all of them failed to deplete both MDSC subsets without affecting other leukocyte subpopulations. In particular, treatment with anti-Gr1 antibodies has been extensively used as an experimental approach to demonstrate the pathogenic effect of MDSCs in infections (34, 47, 69, 70) and cancer (71–73). Anti-Gr1 antibodies can bind to two molecules of the Ly6 superfamily, Ly6G and Ly6C, which are preferentially expressed in granulocytes and monocytes, respectively (74). Therefore, anti-Gr1 treatment could affect the behavior of other myeloid cell subpopulations, such as neutrophils and macrophages. Despite the limitations in the usage of anti-Gr1 antibodies for depleting MDSCs or their subsets in lung tissues, they are a useful research tool when rationally combined with other approaches (34, 47, 69), as performed in these studies.

These depletion strategies allowed us to reduce the presence of MDSCs in lung-infiltrating leukocytes at all infection times studied. PMN-MDSCs were the most affected subset of cells after anti-Gr1 treatment. However, expectedly, a significant reduction in the frequency and number of neutrophils was observed in our study. The morphological proximity between PMN-MDSCs and neutrophils is one of the major challenges in the specific depletion of these cells (75). Despite the decrease in the number of neutrophils caused by anti-Gr1 treatment, higher frequencies of other cell populations, such as macrophages and CD4^+^ T-cells, were detected in anti-Gr1-treated animals compared with those in the IgG2b-treated control group. Moreover, higher frequencies of Th1 and Th17 cells, both of which are associated with disease resistance in PCM (15), were also observed in MDSC-depleted mice at all infection times studied. The increased influx of macrophages to the site of infection was in agreement with the robust Th1 response and CD4^+^IFNγ^+^-producing cells, which function as major macrophage activators, detected in anti-Gr1-treated mice (76). However, the opposite results observed for the CD8^+^ T-population might reflect treatment with anti-Gr1, as the Ly6C epitope was also expressed at low levels on the surface of CD8^+^ T-lymphocytes (77).

Because MDSCs can suppress T-cells and other leukocytes, such as DCs, macrophages, and NK cells (78), we further analyzed the presence of cytokines in lung homogenates. In some aspects, our ELISA data reflected the data obtained from flow cytometric analysis of lung infiltrating cells and other experiments for evaluation of disease progression. Specifically, 2 weeks after infection, we observed higher levels of some cytokines involved in Th1 (TNF-α) and Th17 (IL-6 and IL-17) in the lungs of anti-Gr1-treated mice compared with those in the IgG2b-treated control group. This environment with high levels of protective cytokines in PCM might reflect the depletion of MDSCs, as it promoted a less suppressive environment as determined by the reduced levels of IL-10 and TGF-β, both MDSC-suppressive cytokines (79, 80). However, the high levels of Th1 and Th17 responses (96 h and 2 weeks after infection) and protective cytokines (2 weeks after infection) were not accompanied by reduced fungal loads at 96 h and 2 weeks after infection. The depletion of other protective cell populations in PCM, such as neutrophils (81–83) and CD8^+^ T-lymphocytes (41, 84), probably compromised the initial defense of murine hosts despite increased Th1 and Th17 responses. In fact, in the eighth week of infection, a different scenario was observed. In the chronic phase of infection, the increased Th1 and Th17 responses observed since the early periods of infection resulted in less severe disease, as evidenced by CFU counts and histological analysis. Consequently, the reduction in the levels of IL-6, an inflammatory and Th17-associated cytokine, observed in anti-Gr1-treated animals, likely reflected the lower stimuli promoted by lower fungal loads and regressive disease. In addition, the reduction in the levels of IL-1β might also reflect MDSCs depletion, as this cytokine is also produced by MDSCs, as previously described in the context of *A. fumigatus* and *C. albicans* infections (38).

Despite the reduction in the levels of some pulmonary cytokines, more robust Th1 and Th17 responses were still observed at week 8 after infection. These increased T-cell responses, observed since the acute phase of infection, led to a less severe infection, with decreased fungal burdens in the lungs and liver, resulting in increased survival times. Taken together, our data demonstrated that depletion of Gr1^+^ cells during PCM results in a late, but significant, control of fungal loads in target organs that prevents the expansion of pulmonary lesions and minimizes lung pathology, both of which are possibly associated with high mortality rates in untreated mice. These data provided clear evidence for the negative suppressive effect of MDSCs on pulmonary PCM. Our results were in consistency with those from two recent elegant studies (34, 47). In a murine tuberculosis infection model, the influx of PMN-MDSCs to the pulmonary inflammatory focus was also related to T-cell suppression. Notably, anti-Gr1 therapy, even decreasing the levels of circulating neutrophils and monocytes, prevented the accumulation of MDSCs in the lungs and, therefore, reduced the development of pulmonary granulomatous necrosis and increased the survival of infected mice (34). Li et al. demonstrated that depletion of PMN-MDSCs using anti-Ly6G antibodies in *Cryptococcus neoformans*-infected mice increased Th1 and Th17 responses, leading to a higher survival rate and lower fungal burden than those in control mice (47).

To better demonstrate the deleterious effects of MDSCs in pulmonary PCM and circumvent the broad effect of anti-Gr1 antibodies in several cell subpopulations, we performed a complex cell transfer assay in infected mice. Despite its difficult implementation, this approach yielded important results. The transfer of 2 million MDSCs obtained from *P. brasiliensis-*infected donor mice to recipient mice previously infected for 7 weeks resulted in a significant increase in pulmonary fungal loads compared with that in recipient mice that received only DMEM. Interestingly, an increase in fungal colony counts was not observed in the livers and spleens of recipient mice, a finding that requires further investigation. However, this is likely to be attributed to the adoptive transfer route employed. In fact, MDSCs inoculated through the it. route were expected to remain and exert their suppressive effect in the pulmonary inflammatory focus, as we detected here. The levels of cytokines detected in the lungs of MDSC-recipient mice corroborated the suppressive activity of MDSCs on T-lymphocytes. This adoptive cell transfer resulted in impaired production of IL-2, IL-6, and IL-17. This reduction in Th17-related cytokines might explain the worsening disease in MDSC-recipient mice, as this T-cell subset has been associated with protective immunity in pulmonary PCM (15, 40). Furthermore, our data on the adoptive transfer of MDSCs were in agreement with similar studies demonstrating that the accumulation of MDSCs led to worst disease outcomes in *Staphylococcus aureus* (85) and *Pneumocystis jirovecii* (86) infections. Furthermore, our data indicated that the accumulation of MDSCs at infection sites contributes negatively to host defenses during pulmonary PCM.

Taken together, our data showed that MDSCs abundance in the lungs was linked to more severe disease and associated with weakened Th1 and Th17 protective immune responses. Suppressive molecules, such as IDO-1, IL-10, PD-1, B7-H4, and nitrotyrosine are expressed by MDSCs even in the early stages of *P. brasiliensis* infection. Further studies are necessary to determine the role of each one of these inhibitory molecules in the suppressive activity of MDSCs. However, the immunosuppressive effects of MDSCs on T-lymphocytes were demonstrated *in vitro* and *in vivo*. The immune system contains several components that orchestrate the response against pathogens. Activation of the system triggers proinflammatory and anti-inflammatory responses that, despite being opposites, aim to promote host defenses without causing tissue pathology. The use of this balanced and tightly regulated response allows the immune system to work efficiently, controlling the growth of microorganisms without injuring affected tissues. MDSCs expand and migrate to the inflammatory site of an infection, promoting the control of excessive inflammation. However, among many other mechanisms, this control can be used as a form of immune escape by pathogens. In this context, previous studies on PCM were in agreement with our study. For example, depletion of Treg cells in DEREG mice showed beneficial effects on ongoing PCM because it restored T-cell immunity and reversed disease severity (22). Likewise, increased immunogenic activity of *P. brasiliensis*-infected pDCs was observed when IDO-1-deficient or IDO-1-inhibited pDCs were used in cocultures with lymphocytes. In addition, depletion of pDC-IDO-1^+^ by a specific monoclonal antibody resulted in less severe infection, reduced tissue pathology, and increased the survival time of *P. brasiliensis-*infected mice (16). In both cases, better control of fungal growth was observed after reducing inhibitory mechanisms, such as those mediated by Treg cells or the activity of pDC/IDO-1. Combined with our study, these findings clarified the role of MDSCs in PCM, confirming that immunotherapy strategies for PCM that aim to explore the regulatory mechanisms of the immune system are promising, mainly because of the late diagnosis of the disease, when the infection has been well established.

## Data availability statement

The raw data supporting the conclusions of this article will be made available by the authors, without undue reservation.

## Ethics statement

The studies involving animals were reviewed and approved by the Committee on Animal Experiments of the Federal University of São Paulo (Protocol; 8294230519).

## Author contributions

NP and FL conceived and planned experiments. NP, VK, BB carried out the experiments. NP, VK, FL, VC, BB contributed to the interpretation of the results. NP, FL, VK, VC wrote the paper. NP, FL, VK prepared the figures. FL supervised the project and provided financial support. All authors reviewed the manuscript. All authors contributed to the article and approved the submitted version.
