# Supplementary material for: Myeloid-derived suppressor cells are associated with impaired Th1 and Th17 responses and severe pulmonary paracoccidioidomycosis which is reversed by anti-Gr1 therapy
Source: Front Immunol. 2023 Jan 26;14:1039244. doi: 10.3389/fimmu.2023.1039244 (PMC9909482; doi:10.3389/fimmu.2023.1039244)
Supplement: Supplementary file 1 [file DataSheet_1.pdf]

Supplementary Figure 1

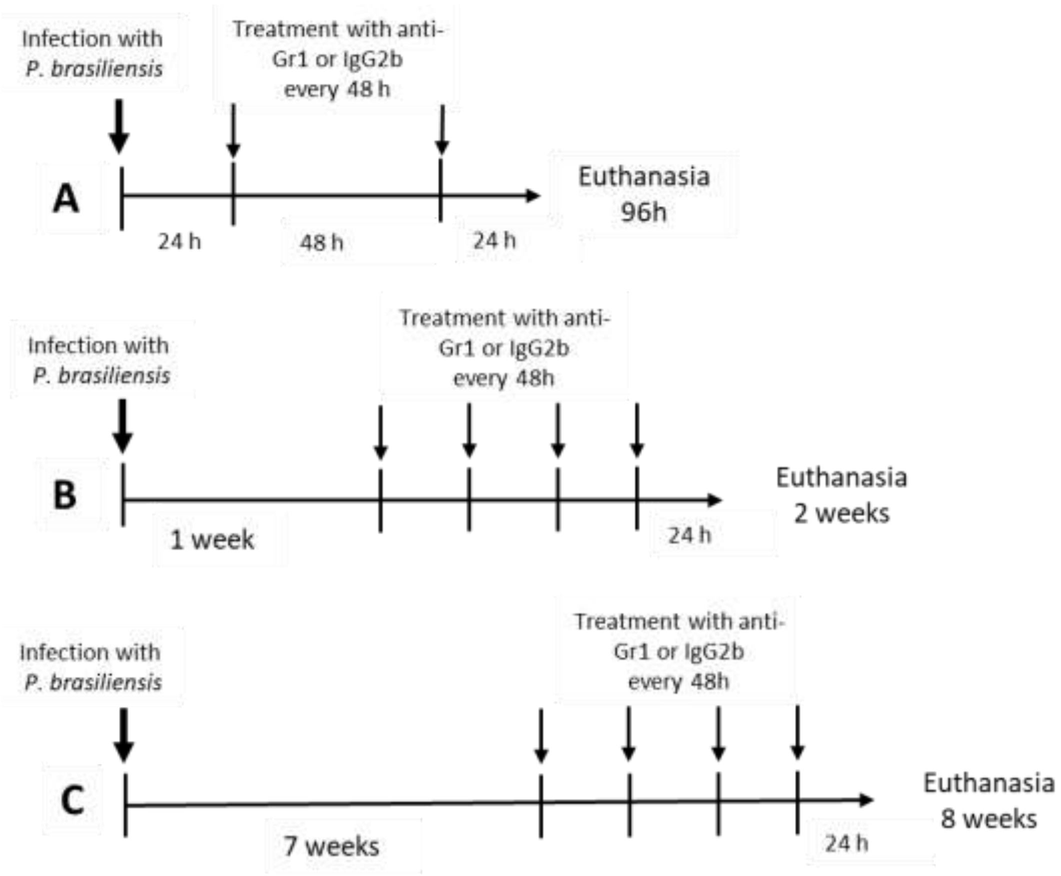

Supplementary Figure 1. The diagram illustrates the anti-Gr1 treatments used to analyze the disease severity and the lung infiltrating leukocytes after infection. C57BL/6 WT mice were infected with 1×10^6 *P. brasiliensis* yeast cells. After 24 h, 1 week, and 7 weeks of infection, a group of mice received intraperitoneal injections of anti-Gr1 antibody, or rat IgG2b isotype control antibody (200 µg/dose). Injections were administered every 48 h. After 96 h, 2 weeks, and 8 weeks of infection, the mice were euthanized. The lung, liver, and spleen were collected to analyze the disease severity, through evaluation of colony-forming unit (CFU) counts and the levels of cytokines by ELISA. The depletion of MDSCs and other leukocytes in the lungs was monitored using flow cytometric analysis.
